# Overexpression of syndecan-1, MUC-1, and putative stem cell markers in breast cancer leptomeningeal metastasis: a cerebrospinal fluid flow cytometry study

**DOI:** 10.1186/s13058-017-0827-4

**Published:** 2017-04-11

**Authors:** Iole Cordone, Serena Masi, Valentina Summa, Mariantonia Carosi, Antonello Vidiri, Alessandra Fabi, Alessia Pasquale, Laura Conti, Immacolata Rosito, Carmine Maria Carapella, Veronica Villani, Andrea Pace

**Affiliations:** 1 Regina Elena National Cancer Institute, Clinical Pathology Division, Via Elio Chianesi 53, 00144 Rome, Italy; 2 Regina Elena National Cancer Institute, Histopathology Department, Via Elio Chianesi 53, 00144 Rome, Italy; 3 Regina Elena National Cancer Institute, Radiology Department, Via Elio Chianesi 53, 00144 Rome, Italy; 4 Regina Elena National Cancer Institute, Medical Oncology Department, Via Elio Chianesi 53, 00144 Rome, Italy; 5 Regina Elena National Cancer Institute, Neuro-Surgery Department, Via Elio Chianesi 53, 00144 Rome, Italy; 6 Regina Elena National Cancer Institute, Neuro-Oncology Division, Via Elio Chianesi 53, 00144 Rome, Italy

**Keywords:** Breast cancer, Leptomeningeal metastasis, Flow cytometry, Circulating tumor cells, Cancer stem cell, Prognosis

## Abstract

**Background:**

Cancer is a mosaic of tumor cell subpopulations, where only a minority is responsible for disease recurrence and cancer invasiveness. We focused on one of the most aggressive circulating tumor cells (CTCs) which, from the primitive tumor, spreads to the central nervous system (CNS), evaluating the expression of prognostic and putative cancer stem cell markers in breast cancer (BC) leptomeningeal metastasis (LM).

**Methods:**

Flow cytometry immunophenotypic analysis of cerebrospinal fluid (CSF) samples (4.5 ml) was performed in 13 consecutive cases of BCLM. Syndecan-1 (CD138), MUC-1 (CD227) CD45, CD34, and the putative cancer stem cell markers CD15, CD24, CD44, and CD133 surface expression were evaluated on CSF floating tumor cells. The tumor-associated leukocyte population was also characterized.

**Results:**

Despite a low absolute cell number (8 cell/μl, range 1–86), the flow cytometry characterization was successfully conducted in all the samples. Syndecan-1 and MUC-1 overexpression was documented on BC cells in all the samples analyzed; CD44, CD24, CD15, and CD133 in 77%, 75%, 70%, and 45% of cases, respectively. A strong syndecan-1 and MUC-1 expression was also documented by immunohistochemistry on primary breast cancer tissues, performed in four patients. The CSF tumor population was flanked by T lymphocytes, with a different immunophenotype between the CSF and peripheral blood samples (*P* ≤ 0.02).

**Conclusions:**

Flow cytometry can be successfully employed for solid tumor LM characterization even in CSF samples with low cell count. This in vivo study documents that CSF floating BC cells overexpress prognostic and putative cancer stem cell biomarkers related to tumor invasiveness, potentially representing a molecular target for circulating tumor cell detection and LM treatment monitoring, as well as a primary target for innovative treatment strategies. The T lymphocyte infiltration, documented in all CSF samples, suggests a possible involvement of the CNS lymphatic system in both lymphoid and cancer cell migration into and out of the meninges, supporting the extension of a new form of cellular immunotherapy to LM. Due to the small number of cases, validation on large cohorts of patients are warranted to confirm these findings and to evaluate the impact and value of these results for diagnosis and management of LM.

**Electronic supplementary material:**

The online version of this article (doi:10.1186/s13058-017-0827-4) contains supplementary material, which is available to authorized users.

## Background

Leptomeningeal metastasis (LM) is a dramatic complication in neuro-oncology and breast cancer (BC) is one of the most common solid tumors to metastasize to the leptomeninges [1]. Although BCLM remains an incurable disease by current therapies, treatments started in an early stage of the disease significantly increase survival [2, 3]. New approaches are dramatically needed to facilitate diagnosis and treatment response monitoring, as well as the identification of new prognostic biomarkers, able to stratify patients according to risk of metastasis and cerebrospinal fluid (CSF) cancer dissemination. Moreover, the identification of biological markers to utilize as a target for treatment will significantly improve tailoring the best strategies for individual patients, enhancing the poor clinical response rate of LM.

According to the tumor stem cell hypothesis, a subset of cells, defined as cancer-initiating cells, has a primary relevance in tumor metastases and cancer recurrence after chemotherapy [4–6]. This subpopulation, residing in a heterogeneous primary tumor, exhibits enhanced invasive properties as well as the ability to grow in anchorage-independent conditions [7, 8]. Isolated from solid biopsies and tumor cell lines, cancer stem cells are currently identified by surface antigen expression using a number of putative stem cell markers including CD15, CD24, CD44, and CD133 [9–13].

An aberrant protein expression can be significantly associated with cancer dissemination and poor prognosis. In BC, MUC-1 (CD227) overexpression has been correlated with cell adhesion inhibition and increased metastatic potential of tumor cells [14–17] while syndecan-1 (CD138), a transmembrane receptor involved in cell-cell adhesion, cell mobility, proliferation and differentiation, has been related to an aggressive phenotype and poor clinical behavior [18–20].

Flow cytometry is a sensitive method for the identification of CSF infiltration in onco-hematology [21–23]. However, studies regarding the role of CSF flow cytometry in solid tumors LM are still limited [24–27].

Focusing on one of the most aggressive BC cell subpopulations that reach the CNS from the primitive tumor, we evaluated, by flow cytometry, the expression of putative prognostic, cell adhesion molecules and cancer stem cell markers on CSF floating tumor cells of patients with BCLM. The tumor-associated population of lymphocytes was also characterized and compared to the peripheral blood immunophenotype.

## Methods

### Patients

Patients with a BC who underwent lumbar puncture for neurological signs and symptoms and a gadolinium-enhanced magnetic resonance strongly suspicion for LM [28] entered the study. The Central Ethics Committee IRCCS Lazio, Section IRCCS I.F.O. approved the study. An informed consent was obtained from all patients.

### Histopathology

Patients were staged according to the International Union Against Cancer Tumor Node Metastasis (UICC-TNM) classification by conventional histology (H&E) and immunohistochemistry (IHC) on formalin-fixed, paraffin-embedded tissue utilizing the following antibodies: estrogen receptor (ER), progesterone receptor (PgR) and human epidermal growth factor receptor 2 (*HER2*) from Dako, Milan, Italy. A subset of cases was also investigated for syndecan-1 (CD138, clone MI15, Dako, Italy) and MUC-1 (CD227, clone HMPV, from BD Pharmingen, San Diego, CA, USA). Immunostaining was performed on 3-micron sections treated with the microwave antigen retrieval system, incubated for 1 hour at room temperature with the primary antibodies and processed by a streptavidin-biotin-enhanced immunoperoxidase technique, according to the manufacturer’s recommendations.

### CSF collection

A total volume of 7 ml (range 1.5–11) of CSF was collected in a tube without any transport medium and processed within 1–3 hours from collection to minimize cell loss. To avoid peripheral blood contamination, the first 0.2–0.4 ml of CSF was discarded before sample collection.

### CSF cell count and morphological analysis

A volume of 1.5 ml (range 1–3) of CSF was utilized for cell count and morphology. Standard cell count was performed by using the Turk reagent and a Nageotte chamber. Morphological examination was performed on cytospin using the Thinprep plus Papanicolaou method [29] by experienced cytopathologists unaware of the flow cytometry analysis. We defined CSF localization as any positive sample by cytology.

### CSF flow cytometry assay

A volume of 4.5 ml (range 1–10) of CSF was utilized for flow cytometry analysis. The CSF was spun at 1500 rpm for 7 minutes, the supernatant fluid was discarded and the cell pellet was suspended in PBS and stained, according to the manufacturer’s recommendations. The following monoclonal antibodies (mAbs) were used: CD15Fitc, CD24Pe, CD34Pe-Cy7, CD44Fitc, CD45PerCP, CD133Pe, CD133APC, CD138Pe, CD138APC, and CD227Fitc. Incubation was performed using the BD Biosciences (San Diego, CA, USA) FACS Lyse and Wash Assistant according to the Duo-Lyse program. Prior to sample acquisition, a flow cell cleaning with distilled water (for 1 to 2 minutes run) was performed to avoid sample carryover. The whole volume of the sample was acquired and analyzed using the FACSCanto II 2L flow cytometer and the FACSDiva software Version 6.1.3 (BD Biosciences). Single-stained cellular controls and BD FACS™ 7-color setup beads were used to adjust detector voltage, to set fluorescence compensation, and to monitor instrument performance. Positive and negative markers on different subpopulations were used as an internal isotype control. Tumor cell markers were repeated up to three times in a proportion of cases. Moreover, syndecan-1 expression was evaluated using different fluorochromes in six cases. The BC phenotype was evaluated on CSF floating cells by gating on the CD45 negative versus side scatter (SSC) large cells (lymphocytes expressing bright CD45). The population of CSF lymphocytes was characterized according to the following mAbs: CD3Fitc, CD56Pe, CD56APC, CD45PerCP, CD4PE-Cy7, CD19APC, CD19APC-Cy7, and CD8APC-Cy7. The CD4 and CD8 subsets were evaluated as the percentage of CD3-positive T lymphocytes. Monocytes were identified using the CD4 dim and CD14APC-Cy7. All mAbs were from BD Biosciences, except CD133 from Miltenyi Biotec, Bergisch Gladbach, Germany and CD138Pe (Clone B-A38) from Beckman Coulter, Brea, CA, USA. Data are presented as the percentage of positive cells, evaluated on the CD45-negative/SSC large cells for BC cell analysis and on the CD45-positive population for the tumor-associated leukocytes. The mean fluorescence intensity (MFI) ratio for the syndecan-1 and MUC-1 antigens was calculated by comparison with negative control. The lymphocyte characterization was also conducted on corresponding peripheral blood (PB) samples.

### Statistical analysis

Wilcoxon rank-sum test was conducted to evaluate the different distribution between CSF and PB lymphoid subpopulations. The test was two-sided with a *p* value of 0.05 indicating a statistically significant difference.

## Results

### Patient characteristics

Thirteen patients with a BC who underwent lumbar puncture for clinical suspicion of LM at the Regina Elena National Cancer Institute were enrolled. All patients were female with a median age of 50 years (range 44–69). In all cases neurological signs, symptoms, and a gadolinium-enhanced magnetic resonance suspicion for LM was documented.

### Histopathology

**Fig. 1:**
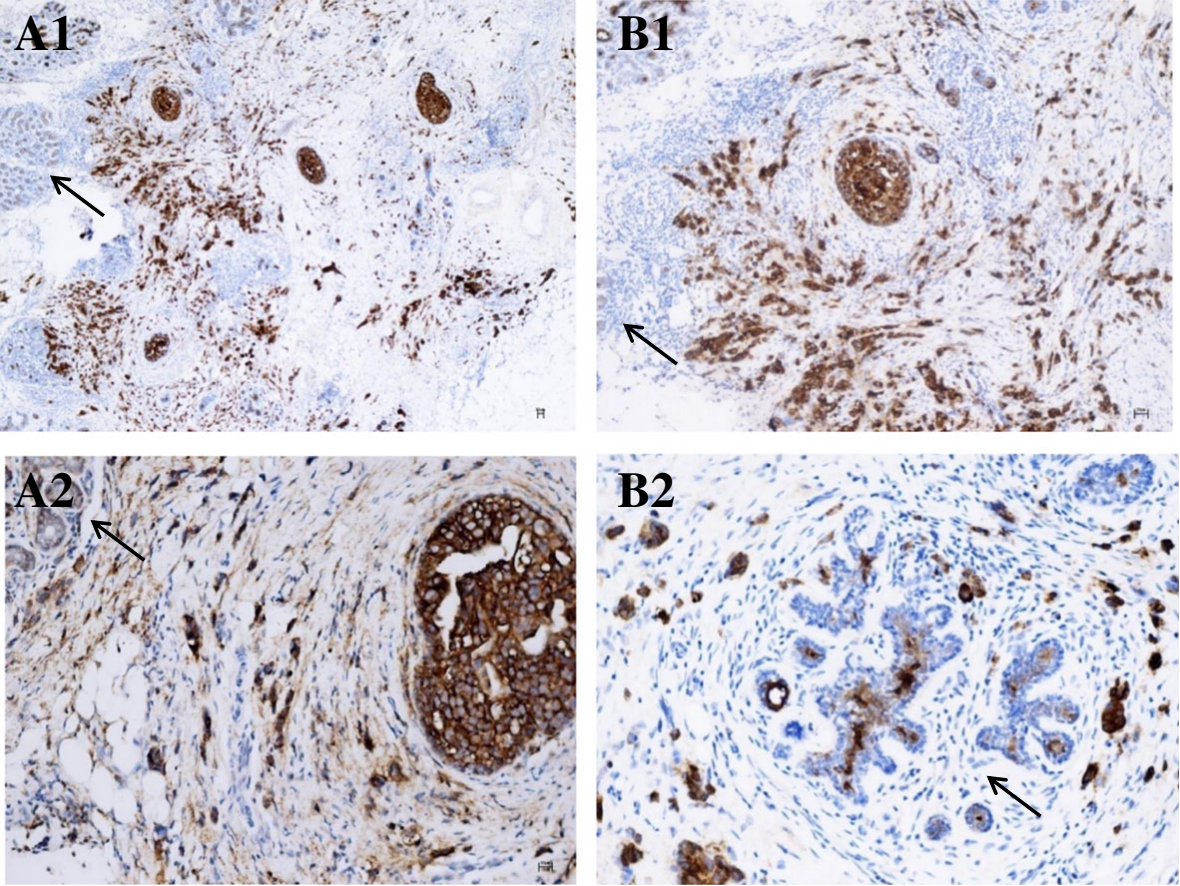
Primary breast cancer tissue immunostaining of patients with leptomeningeal metastasis. Breast cancer cells are syndecan-1 (CD138) (**a1** and **a2**) and MUC-1 (CD227) (**b1** and **b2**) strongly positive (*intense brown staining* of both in situ and infiltrating tumor cells). Non-neoplastic breast epithelium (*arrow*) shows glandular architecture with weak staining for both syndecan-1 and MUC-1 antibodies

The histological and IHC characteristics of the primary BC tumor are presented in Table 1. In three patients, BC was diagnosed in other centers and detailed histological data were not available; these three outpatients were referred to the Regina Elena National Cancer Institute Neuro-Oncology Division for diagnosis and treatment of LM clinical symptoms. An infiltrating BC carcinoma was documented in all the cases analyzed by histopathology. MUC-1 and syndecan-1 IHC staining of breast primary carcinoma tissues, performed in four patients (number 6, 9, 11, and 13), revealed strong brown staining of *in situ *and infiltrating breast carcinoma cells with both MUC-1 and syndecan-1 antibodies. Intense staining appears to be both cytoplasmic and on the cell surface. By contrast, non-neoplastic breast epithelium from patients with breast cancer showed clear glandular architecture with weak staining with MUC-1 and syndecan-1 antibodies. Tumor-infiltrating lymphocytes were negative for both antibodies (Fig. 1).

**Table 1:** Histological and immunohistochemical staining of the primary breast cancer tissue from patients with leptomeningeal metastasis

Case number	Histology	pTNM	ER	PgR	*HER2*
1	na	na	na	na	na
2	Infiltrating ductal carcinoma	na	pos	pos	neg
3	Infiltrating ductal carcinoma	pTc1, N0, stage 1	neg	neg	neg
4	Infiltrating lobular carcinoma	na	neg	pos	pos
5	na	na	na	na	na
6	Infiltrating ductal carcinoma	pT4b, N3a, M1 (UICC 2002)	40%	70%	neg
7	Infiltrating lobular carcinoma	pT2, pN3a, M0	75%	100%	pos
8	na	na	na	na	na
9	Infiltrating lobular carcinoma	pT1c, N1bi, Mx (UICC 1997)	30%	30%	neg
10	Infiltrating ductal carcinoma	na	pos	pos	neg
11	Infiltrating lobular multifocal	pT1c (m); pN1biv; Mx (UICC 1997)	20%	20%	pos
12	Infiltrating ductal carcinoma	na	neg	neg	neg
13	Infiltrating ductal carcinoma	pT1c(m), N2, Mx (UICC 2002)	neg	40%	pos

*pTNM* pathological tumor-node-metastasis stage, *ER* estrogen receptor, *PgR* progesterone receptor, *HER2* human epidermal growth factor receptor, *na* not available, *pos* positive, *neg* negative

### CSF cell count and cytology

The CSF samples had a median cell count of 8 cell/μl (range 1–86) with an increased leukocyte count (>3/mm^3^) in 61% of cases (Table 2). A diagnosis of BCLM was documented in all 13 cases by cytological identification of malignant cells. The cancer cell population was sided by reactive lymphocytes and monocytes in all samples. A peripheral blood contamination was documented in two cases by a prevalence of red blood cells and neutrophil granulocytes between the cancer cells (Table 3; case number 1 and number 13.).

**Table 2:** CSF flow cytometry characterization of cancer floating cells in 13 cases of breast cancer leptomeningeal metastasis

Case number	CSF cellularity (cells/μl)	Number of events analyzed	Percentage of BC cells (CD45-negative)	Percentage of positive cells within the CD45-negative BC population					
				CD15%	CD138%	CD227%	CD24%	CD44%	CD133%
1	32	181,000	9	nd	82	nd	nd	nd	nd
2	1	2887	47	nd	99	nd	nd	nd	nd
3	86	132,260	43	24	96	nd	98	94	96
4	66	12,817	96	51	97	nd	nd	nd	neg
5	2	6078	4	75	80	nd	nd	nd	93
6	50	45,134	20	neg	91	93	94	97	neg
7	1	4612	20	66	54	97	90	88	59
8	2	1183	35	nd	80	94	nd	87	80
9	30	24,300	47	neg	56	82	neg	neg	neg
10	17	29,798	40	92	98	96	77	96	neg
11	1	1293	60	12	93	96	100	99	neg
12	8	3550	45	98	89	97	neg	56	neg
13	8	8455	36	neg	66	88	89	neg	88

Breast cancer marker expression is reported as the percentage of positive cells within the CD45-negative/side-scatter large population

*CSF* cerebrospinal fluid, *BC* breast cancer, *nd* not done, *neg* negative

**Table 3:** CSF flow cytometry characterization of the infiltrating leukocyte in 13 cases of breast cancer leptomeningeal metastasis

Case number	CD45-positive cells (%)	Leucocytes distribution among the CD45+ population			CSF lymphocytes subpopulation						
		% lymphocytes (CD45 SSC)	% monocytes (CD14+)	% neutrophils (CD15+)	% CD3	% CD56	% CD3/CD4	% CD3/CD8	% CD3/CD56	T4/T8 ratio	% CD19
1	91	16	6	78	93	9	67	32	4	2.09	1
2	53	42	38	20	94	8	58	42	9	1.38	1
3	57	85	12	3	92	9	63	30	4	2.1	0
4	4	70	18	12	94	5	58	41	1	1.41	0
5	96	84	10	6	97	6	63	36	5	1.75	1
6	80	80	19	1	96	3	68	30	1	2.26	1
7	80	64	29	7	92	10	58	40	4	1.45	0
8	65	74	22	4	98	24	68	33	30	2.06	0
9	53	55	40	5	85	10	47	51	5	0.9	0
10	60	40	57	3	87	19	47	53	7	0.9	3
11	40	85	12	3	92	6	45	52	3	0.86	0
12	55	80	12	8	83	14	58	43	4	1.34	3
13	64	6	12	82	75	5	59	36	2	1.6	0

The CSF lymphocytes immunophenotype is reported as percentage of positive cells within the lymphoid population, identified as CD45-strong/intermediate side-scatter signals

*CSF* cerebrospinal fluid, *BC* breast cancer

### Flow cytometry assessment

#### CSF samples

Lumbar puncture yielded adequate material for flow cytometry analysis in all the cases. Despite the low CSF absolute cell number, a median of 8455 (range 1183–181,000) evaluable cells were analyzed. The CD45-negative large cancer cells (35%, range 4–96) were sided by CD45-positive leukocytes in all CSF samples (Table 3).

#### Breast cancer markers expression

**Fig. 2:**
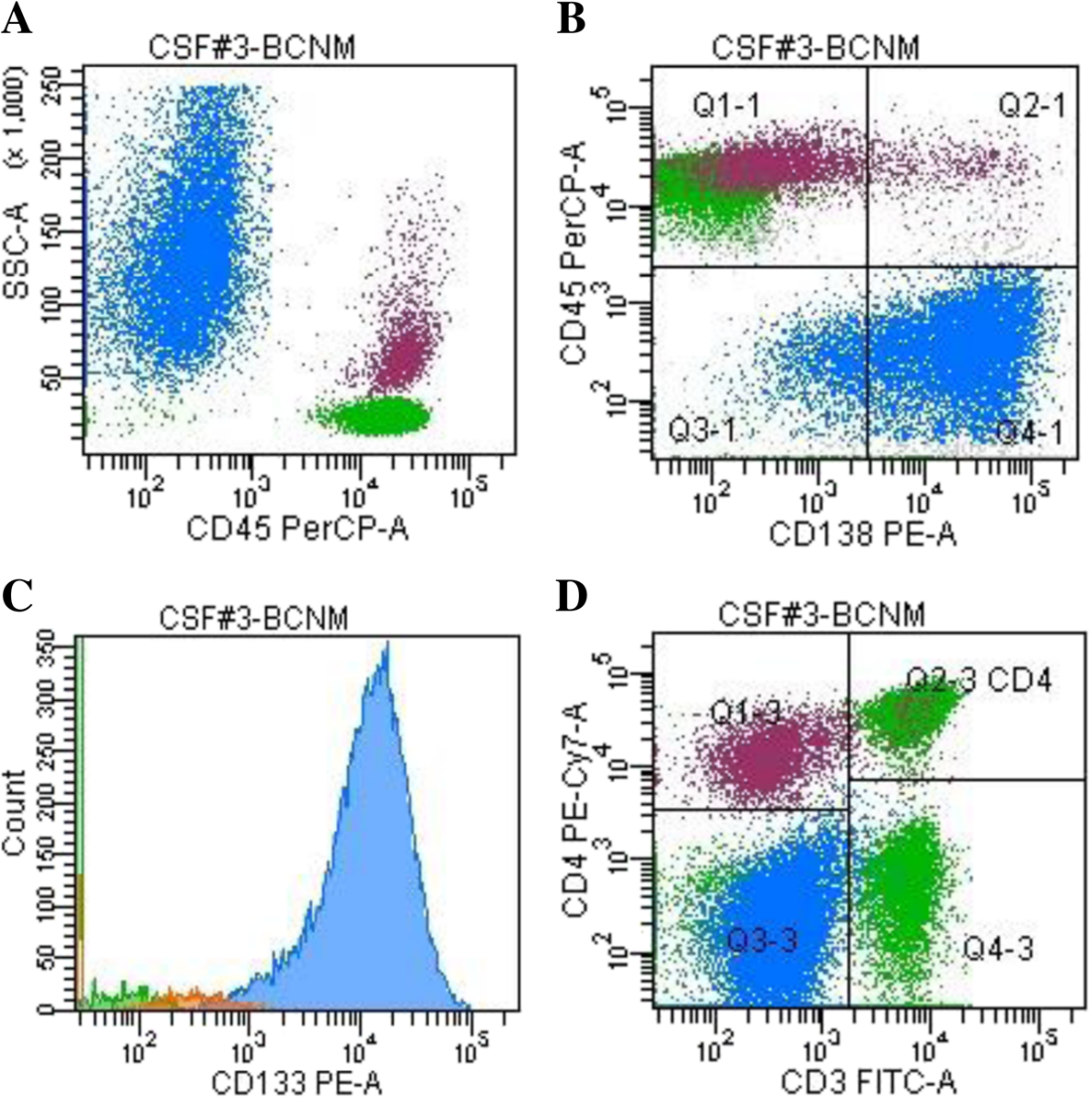
Cerebrospinal fluid (CSF) flow cytometry characterization in breast cancer leptomeningeal metastasis. Breast cancer cells (*blue*) are CD45 negative (**a**), CD138 (**b**), and CD133 (**c**) positive, sided by CD45-positive T lymphocytes (*green*) and monocytes (*purple*) (**a** and **d**)

BC cells were identified by gating on CD45 negative *versus* SSC. BC cells were CD138 and CD227 bright positive in all the cases analyzed, with a median of 83% (54–99) and 93% (82–97) positive tumor cells, respectively (Fig. 2) (Table 2). The MFI ratio was 442 (range 128–599) for CD138 and 566 (range 391–1715) for CD227.

Regarding the putative cancer stem-cell marker expression, seven of ten CSF samples (70%) were CD15 positive with more than 20% CD15+/CD45neg cells in all but one case (Table 2; case number 11). BC cells were CD24 positive in 6/8 (75%), CD44 positive in 7/9 (78%) and CD133 positive in 5/11 cases (45%) respectively (Table 2). Additional file 1: Figure S1. A number of positive BC markers were repeated up to three times in ten samples, confirming the previous acquisition in all the cases. Moreover, syndecan-1 expression was confirmed using different fluorochromes in six cases. CD34-positive cells were not found in any of the samples analyzed. No CD45-negative cells were documented in 20 CSF samples evaluated for hematological malignancies, used as a negative control (data not shown).

#### Phenotype of tumor-associated leukocytes

Beside the BC cells, a tumor-associated population of CD45-positive leukocytes (60%; range 4–96%) was identified in all CSF samples, represented by lymphocytes (61%; range 6–85%) and monocytes (CD14 positive = 18%; range 6–57%) (Fig. 2 a and d). Blood contamination was observed in two cases where a majority of neutrophil granulocytes (CD45/CD15 bright = 78% and 82% respectively) was documented (case number 1 and number 13; Table 3). The lymphoid population was represented by CD3-positive T cells with a prevalence of CD4-positive lymphocytes in 10/13 (77%) cases and 9% of CD56-positive cells (range 3–24). Rare CD19-positive B cells were identified. No BC cells were CD3, CD4, CD8, CD56, CD19, CD45 or CD14 positive.

#### Immunophenotype of peripheral blood lymphocytes

**Fig. 3:**
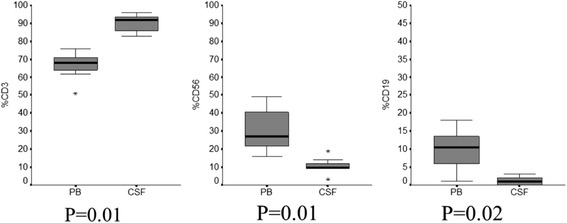
Flow cytometry characterization of cerebrospinal fluid (CSF) and peripheral blood (PB) lymphocytes in patients with breast cancer leptomeningeal metastasis. Wilcoxon rank-sum test documents a significant different distribution between CSF and PB lymphoid subpopulations

The peripheral blood lymphocyte subset was evaluated in 8/13 cases and compared to the CSF lymphoid subpopulations. The absolute number of lymphocytes was 1300 cell/μl (range 300–3600). A population of CD3-positive cells (68%, range 51–76) with a CD4/CD8 ratio of 1.14 (range 0.86–2.26), sided by CD56 positive (27%; range 16–49%) and CD19 positive (10%; range 1–18%) lymphocytes was documented. A different distribution of CD3, CD56 and CD19 lymphoid subpopulations was observed comparing peripheral blood and corresponding CSF samples (*P* ≤ 0.02) (Fig. 3 and Additional file 2: Table S1).

## Discussion

Metastasis is the main reason for cancer-related mortality and currently there are no established biomarkers that stratify BC patients at risk for LM [30–32]. Focusing on one of the most aggressive CTC type, which from the primitive tumor spreads to the CNS, we documented that CSF floating cancer cells overexpress syndecan-1, MUC-1 and, in a proportion of cases, the putative stem-cell markers CD15, CD24, CD44, and CD133 in BCLM.

This is a pilot study, conducted on a limited number of patients, focusing on a subset of rare samples. In ten cases, CSF staining was repeated for some markers, confirming the previous result; moreover, syndecan-1 expression was also confirmed using different fluorochromes in six cases. Although there was a high rate of repeatability observed in our cases, reproducibility, determination of the limits of detection and quantification need to be confirmed in a large cohort of patients.

Surface syndecan-1 and MUC-1 were brightly overexpressed, with a high MFI, on BC cells in all the CSF samples analyzed by flow cytometry. Analysis of MUC-1 and syndecan-1 was carried out by IHC on breast primary carcinoma tissues in four patients. As observed by flow cytometry on CSF floating cancer cells, a strong expression was documented by IHC on breast primary carcinoma tissues, while a weak/negative staining was observed on non-neoplastic breast epithelium. Validation on large cohorts of patients with BC and LM and with BC without LM is required. Moreover, multicenter studies, comparing the immunophenotype of primary tumor and CSF floating cancer cell in LM, are warranted to confirm these data and to investigate the clinical-pathological relevance.

High syndecan-1 is an independent marker of poor prognosis [18–20]. The prospective measurement of syndecan-1 on breast biopsies at diagnosis can potentially contribute to patient risk stratification toward tailored anti-cancer therapies. Highly expressed on neoplastic plasma cells, syndecan-1 has shown to be a viable target for myeloma therapy [33, 34]. Our results suggest syndecan-1 as a potential molecular therapeutic target for innovative antibody-based treatment strategy in BC.

One of the most challenging aims in cancer research is the identification and characterization of the cancer stem cell subset. Anchorage-independent culture of tumor cells enriches cultures from cancer-initiating cells; however the expression of putative cancer stem cell markers can be significantly influenced by in vitro culture conditions [35]^.^ The nature provides a perfect model of anchorage-independent tumor growth in LM. Thereafter, hypothesizing that a BC cell could take advantage from a cancer stem cell phenotype for CSF infiltration, we investigated the expression of a number of putative cancer stem cell markers on BC floating cells, documenting a stem cell-like phenotype, CD15, CD44, CD24, and CD133 positive, in a proportion of cases. Our in vivo approach avoided all the possible phenotypic changes related to the in vitro culture conditions, providing evidence of the potential involvement of a stem cell-like phenotype in the mechanism of CSF invasion, highlighting a number of surface markers as potential targets for inhibition of cancer dissemination.

CD15 (Lewis x) is overexpressed on various cancers and it has been reported as a cell adhesion molecule with a key role in non-CNS cancer metastasis [36, 37]. Lewis x increased expression correlates with poor survival in colorectal and prostate carcinomas [38, 39] and has been identified as a potential cancer stem cell marker in glioma spheroids [40]. In vitro studies have shown CD15 to be involved in the adhesion of MCF-7 human breast cancer cells to human umbilical endothelial cells (HUVEC) and that the anti-Lewis x mAb MCS-1 inhibits this interaction and efficiently lyses BC cells bound to HUVEC without damaging endothelial cells [41]. More recently, a crucial role of CD15 in cancer cell-endothelium adhesion for non-small cell lung cancer cell extravasation to the brain has been reported [42]. Our in vivo study documents, for the first time, the CD15 overexpression in CSF cancer floating cells of BCLM samples. This data supports the interaction between BC cells and endothelium through Lewis x epitopes as a mechanism for CSF invasion in LM. These results are consistent with previous studies that referred to the correlation between elevated levels of CD15 and brain metastasis in different types of non-CNS cancers [43, 44] supporting CD15 as a putative marker of poor prognosis, involved in the aggressive behavior and tumor recurrence, and a possible target for prevention of brain metastases. The adhesion of cancer cells to endothelium can be significantly decreased by absence of CD15 and CD15 immunoblocking [42]. Our study supports the hypothesis of Lexis x as a potential target for inhibition of BC metastasis utilizing anti-Lewis x immunoblocking.

Emerging evidence suggests that a small subpopulation of tumor cells, identified by the CD44^+^/CD24^−/low^ cancer stem cell markers expression in breast cancer tissue, have strong abilities of self-renewal and are responsible for tumor aggressiveness, recurrence, metastasis, and therapeutic resistance [45–48]. CD44-positive and CD24-positive cells have been proposed as predictors of prognosis and treatment response in BC, with clinical implications for cancer treatment because of their role in chemoresistance [49]. The results of the present study document the CD24 and CD44 overexpression in CSF cancer floating cells of BC patients with LM. This finding supports a possible mechanism of positive selection of the stem cell-like phenotype in the genesis of CSF infiltration.

The cancer stem cell marker CD133 has been associated with the presence of adverse biomarkers and subtypes, with a potential predictive role in clinical management of BC patients [50]. Moreover, a close association between CD133 expression and tumor angiogenesis has been reported in invasive breast cancer [51]. Despite the small number of cases, our study documents, in a proportion of case, CD133 overexpression on CSF floating cells of BC patients, supporting the role of CD133 as a marker of poor prognosis.

Biomarkers able to identify patients at risk of undergoing metastatic spread are urgently needed to develop early detection methods and more effective treatment strategies. The peripheral blood CTC detection and enumeration holds promise to provide information on tumor burden and dissemination, disease progression, and treatment response monitoring. Detection of these rare cells on a background of millions of leukocytes poses a great challenge, and several techniques are currently being considered by the international scientific community [52–56]. Our data potentially add a number of surface markers to be tested for CTCs search, flanking the epithelial cell antigen molecule (EpCAM)-positive strategies utilized in epithelial primary tumors [57].

CSF cytology, the diagnostic gold standard for LM identification, is a procedure with considerable limitations regarding sensitivity and no molecular characterization regarding specificity, with a reported false-negative rate of up to 60% and a leukocyte count <4 cell μL in about 30% of cases [58]. Thereafter, patients with low-volume disease, who are likely to benefit more from treatment, are more likely to be false negative. Magnetic resonance imaging with gadolinium enhancement is the technique of choice to evaluate patients with suspected LM [28, 59] and, with suggestive radiological evidence of LM, treatment is warranted despite persistently negative CSF cytology [60]. Thereafter, treatment response is evaluated by clinical improvement of neurological signs and symptoms [61]. Flow cytometry is a proven valuable diagnostic tool in hematological CSF infiltration detection [21, 22, 62]. We have recently documented that flow cytometry can discriminate between reactive and neoplastic plasma cells in CSF samples with very low cell counts, confirming it to be significantly more sensitive than standard approaches [23]. However, so far, only limited published experiences about the use of flow cytometry for the identification of solid tumor LM are reported [24–27]. This study supports the role of flow cytometry as a simple and reliable technique for LM identification, including CSF samples with normal cell count. In fact, despite the CSF low volume (median 4.5 m) and low cell count (median 8 cell/μL), flow cytometry characterization was successfully conducted in all samples, including the five patients (40%) with a cell count <3 ml. More recently, EpCAM-based flow cytometry assay has shown to represent a sensitive approach for the diagnosis of LM in patients with primary epithelial tumors [27]. We documented syndecan-1 and MUC-1 overexpression on BC cells in the cases analyzed. These markers potentially represent innovative and powerful tools for BCLM diagnosis by flow cytometry, able to reduce the proportion of diagnostic failure of LM, to prevent multiple lumbar punctures, and reduce treatment delay. Moreover, their potential role as diagnostic markers for LM of primary epithelial tumors other than BC deserves to be evaluated. Finally, morphology being a very poor technique in minimal residual disease evaluation, they could represent new tools for treatment monitoring of solid tumor LM by flow cytometry, particularly in CSF samples with low cell count.

The tumor inflammatory response is involved in both cancer growth inhibitions as well as in cancer invasiveness [63–67]. However, little is known about the tumor-associated population in LM [25, 26]. The long-held dogma of the lymphatic system absence in the CNS has been recently disproved. In searching for meningeal T cell gateways, functional lymphatic vessels lining the dural sinuses have been discovered. These structures, expressing the molecular hallmarks of lymphatic endothelial cells, are able to carry both fluid and immune cells from the CSF and are connected to the deep cervical lymph nodes [68]. Regarding the immune cell migration into CNS, we have recently documented evidence of an active mechanism of reactive CD8 T lymphocytes migration in primary brain lymphomas [69]. Beside BC cells, infiltrating T lymphocytes and monocytes were documented in all CSF samples of BCLM, with a significant difference in lymphoid immunophenotype between CSF and PB (*P* ≤ 0.02). This finding supports a lymphocytes subpopulation selection in LM, suggesting a possible involvement of the meningeal lymphatic network in both lymphoid and cancer cell migration into the meninges as a potentially alternative route to the cardiovascular system. In two cases, a prevalence of red blood cells and neutrophil granulocytes between cancer cells was documented, due to PB contamination of the lumbar puncture. In all the cases without PB contamination, neutrophils were not identified, highlighting the importance of excluding the first drops of sample from the collection in order to obtain a reliable evaluation of the CSF leukocyte population. The field of cancer immunotherapy has been re-energized by the application of chimeric antigen receptor (CAR) T cell therapy in cancers [70]. Cell surface antigens can serve as target for tumor rejection. More recently, CAR that recognized cancer-associated Tn-glycoform of MUC-1 has been developed, with target-specific cytotoxicity and tumor growth control in xenograft models of T cell leukemia and pancreatic cancer [71]. A strong MUC-1 expression in CSF floating BC cells of patients with LM was documented in this study. Engineered CAR T cells directed against MUC-1 could potentially represent a rationale for the investigation, in preclinical models, of cellular immunotherapy in LM, for future possible designs of immune-based cancer therapies.

## Conclusions

Overexpression of syndecan-1, MUC-1, and the putative cancer stem cell markers CD15, CD24, CD44, and CD133 has been documented on CSF floating cancer cells of BC patients with LM. This is an exploratory analysis. These results and their value for diagnosis and management of BCLM need validation in large cohorts of patients. Further studies are necessary to determine the sensitivity and specificity of the technique and recommend the diagnostic use of flow cytometry next to cytology in CSF samples of patients clinically suspected for LM. Moreover, further research regarding the promising role of flow cytometry in CSF treatment monitoring need to be performed. Studies investigating the role of the surface markers here identified as putative prognostic biomarkers for tumor invasiveness and CNS involvement, molecular targets in CTC detection, as well as primary targets for innovative and selective treatment strategies are promising research topics. New forms of cellular immunotherapy for brain metastasis could take advantage from the infiltrating population of T lymphocytes and monocytes, very much represented in LM.

## Additional files

Additional file 1: Figure S1. Flow cytometry analysis of cerebospinal fluid (CSF) samples of patients with breast cancer leptomeningeal metastasis. Representative dot plot and histogram for CSF breast cancer cells and tumor-associated leukocytes. (PPTX 2013 kb)

Additional file 2: Table S1. Flow cytometry characterization of cerebrospinal fluid (CSF) and peripheral blood (PB) lymphocytes in patients with breast cancer leptomeningeal metastasis. (XLSX 11 kb)

## Abbreviations

BC: Breast cancer
BCLM: Breast cancer leptomeningeal metastasis
CAR: chimeric antigen receptor
CNS: Central nervous system
CSF: Cerebrospinal fluid
CTC: Circulating tumor cells
EpCAM: Epithelial cell antigen molecule
ER: Estrogen receptor
*HER2*: Human epidermal growth factor receptor
HUVEC: Human umbilical vein endothelial cells
IHC: Immunohistochemistry
LM: Leptomeningeal metastasis
mAbs: Monoclonal antibodies
MFI: Mean fluorescence intensity
na: Not available
nd: Not done
neg: Negative
PB: Peripheral blood
PgR: Progesterone receptor
pos: Positive
pTNM: Pathological tumor-node-metastasis stage: SSC, Side scatter
