# Supplementary material for: Overexpression of syndecan-1, MUC-1, and putative stem cell markers in breast cancer leptomeningeal metastasis: a cerebrospinal fluid flow cytometry study
Source: Breast Cancer Res. 2017 Apr 11;19:46. doi: 10.1186/s13058-017-0827-4 (PMC5387324; doi:10.1186/s13058-017-0827-4)
Supplement: Supplementary file 1 — Flow cytometry analysis of cerebospinal fluid (CSF) samples of patients with breast cancer leptomeningeal metastasis. Representative dot plot and histogram for CSF breast cancer cells and tumor-associated leukocytes. (PPTX 2013 kb) [file 13058_2017_827_MOESM1_ESM.pptx]

## Slide 1
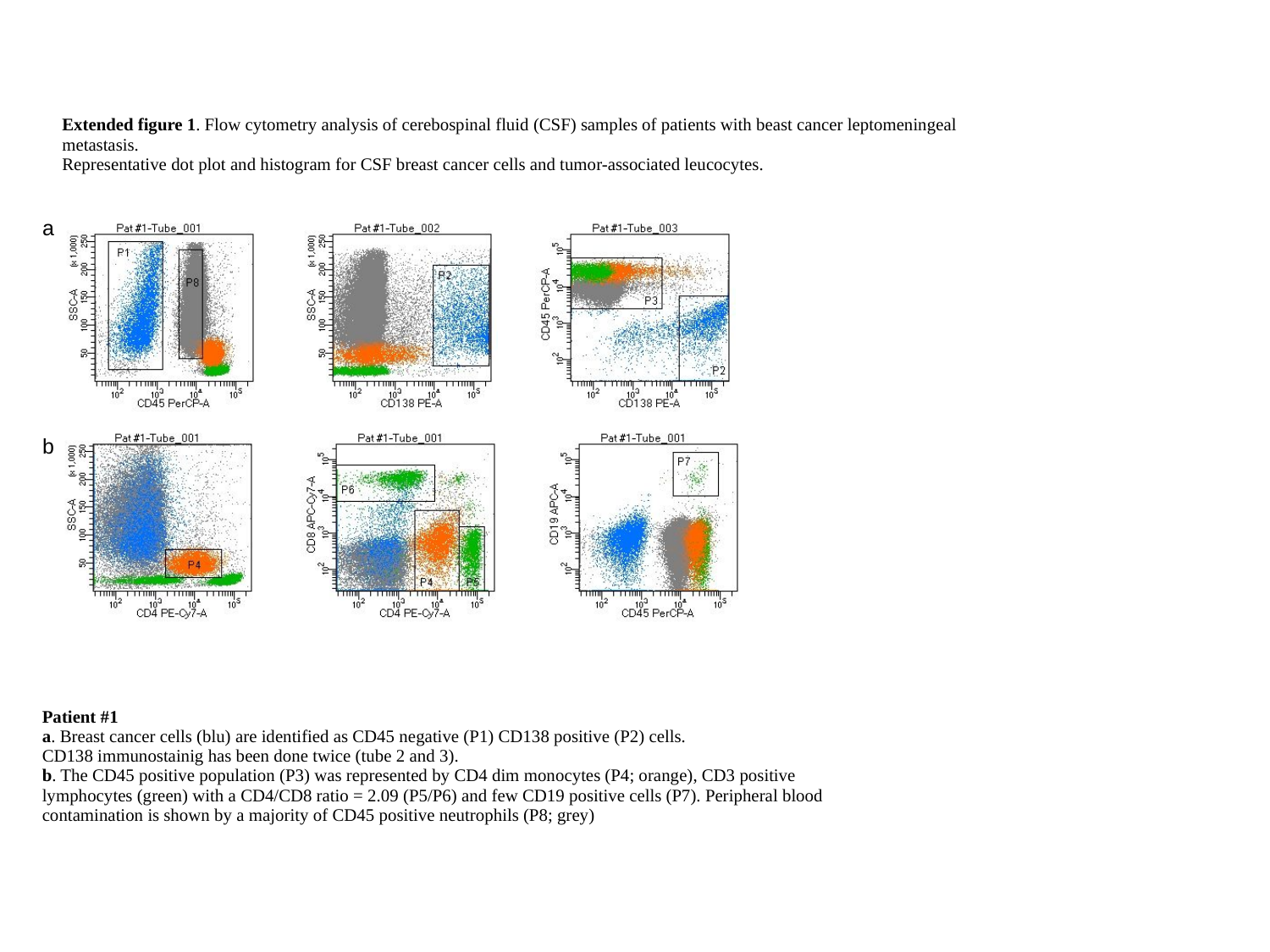

Extended figure 1. Flow cytometry analysis of cerebospinal fluid (CSF) samples of patients with beast cancer leptomeningeal metastasis.
Representative dot plot and histogram for CSF breast cancer cells and tumor-associated leucocytes.
a
b
Patient #1
a. Breast cancer cells (blu) are identified as CD45 negative (P1) CD138 positive (P2) cells.
CD138 immunostainig has been done twice (tube 2 and 3).
b. The CD45 positive population (P3) was represented by CD4 dim monocytes (P4; orange), CD3 positive lymphocytes (green) with a CD4/CD8 ratio = 2.09 (P5/P6) and few CD19 positive cells (P7). Peripheral blood contamination is shown by a majority of CD45 positive neutrophils (P8; grey)

## Slide 2
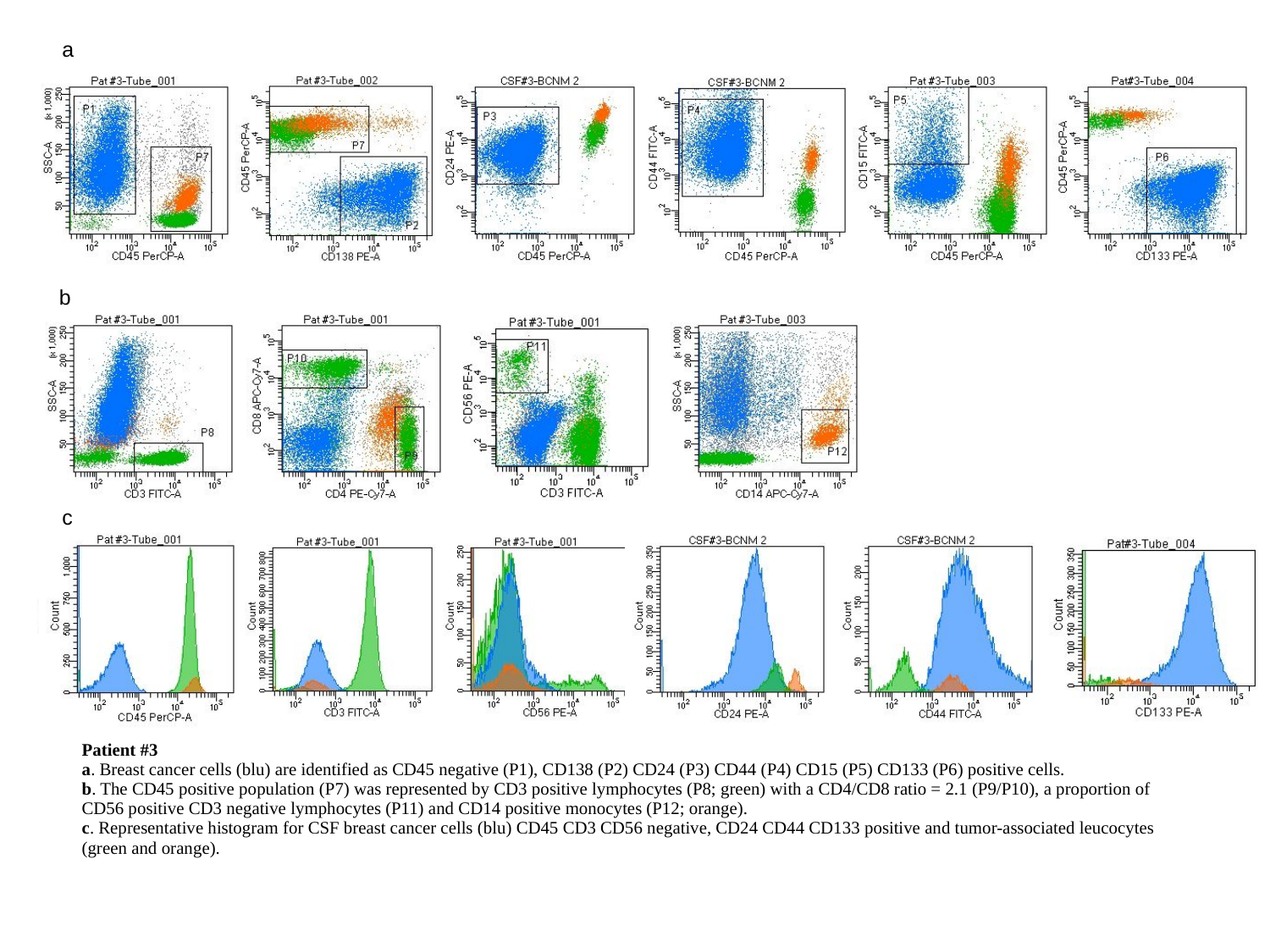

a
b
c
Patient #3
a. Breast cancer cells (blu) are identified as CD45 negative (P1), CD138 (P2) CD24 (P3) CD44 (P4) CD15 (P5) CD133 (P6) positive cells.
b. The CD45 positive population (P7) was represented by CD3 positive lymphocytes (P8; green) with a CD4/CD8 ratio = 2.1 (P9/P10), a proportion of CD56 positive CD3 negative lymphocytes (P11) and CD14 positive monocytes (P12; orange).
c. Representative histogram for CSF breast cancer cells (blu) CD45 CD3 CD56 negative, CD24 CD44 CD133 positive and tumor-associated leucocytes (green and orange).

## Slide 3
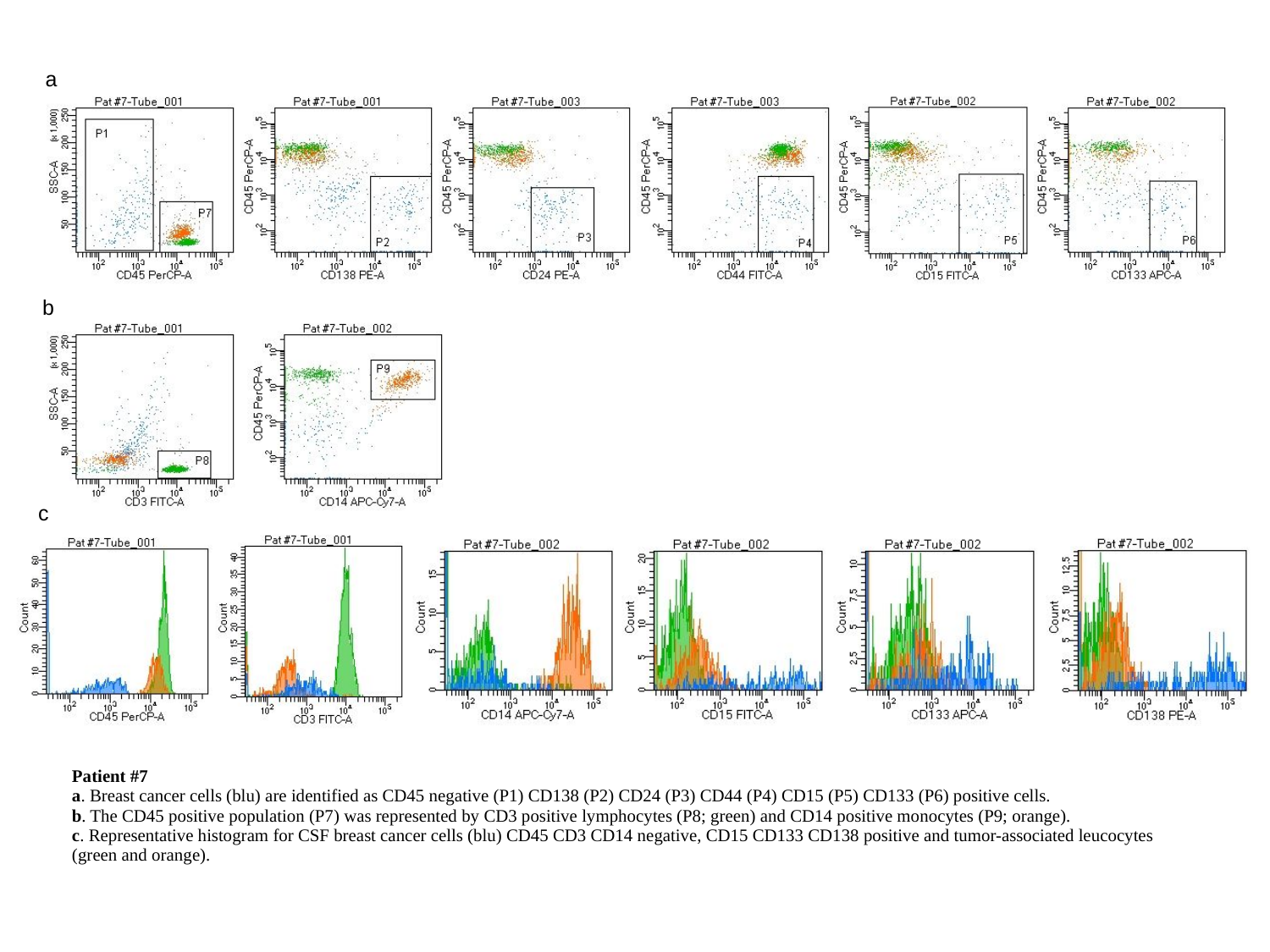

a
b
c
Patient #7
a. Breast cancer cells (blu) are identified as CD45 negative (P1) CD138 (P2) CD24 (P3) CD44 (P4) CD15 (P5) CD133 (P6) positive cells.
b. The CD45 positive population (P7) was represented by CD3 positive lymphocytes (P8; green) and CD14 positive monocytes (P9; orange).
c. Representative histogram for CSF breast cancer cells (blu) CD45 CD3 CD14 negative, CD15 CD133 CD138 positive and tumor-associated leucocytes (green and orange).

## Slide 4
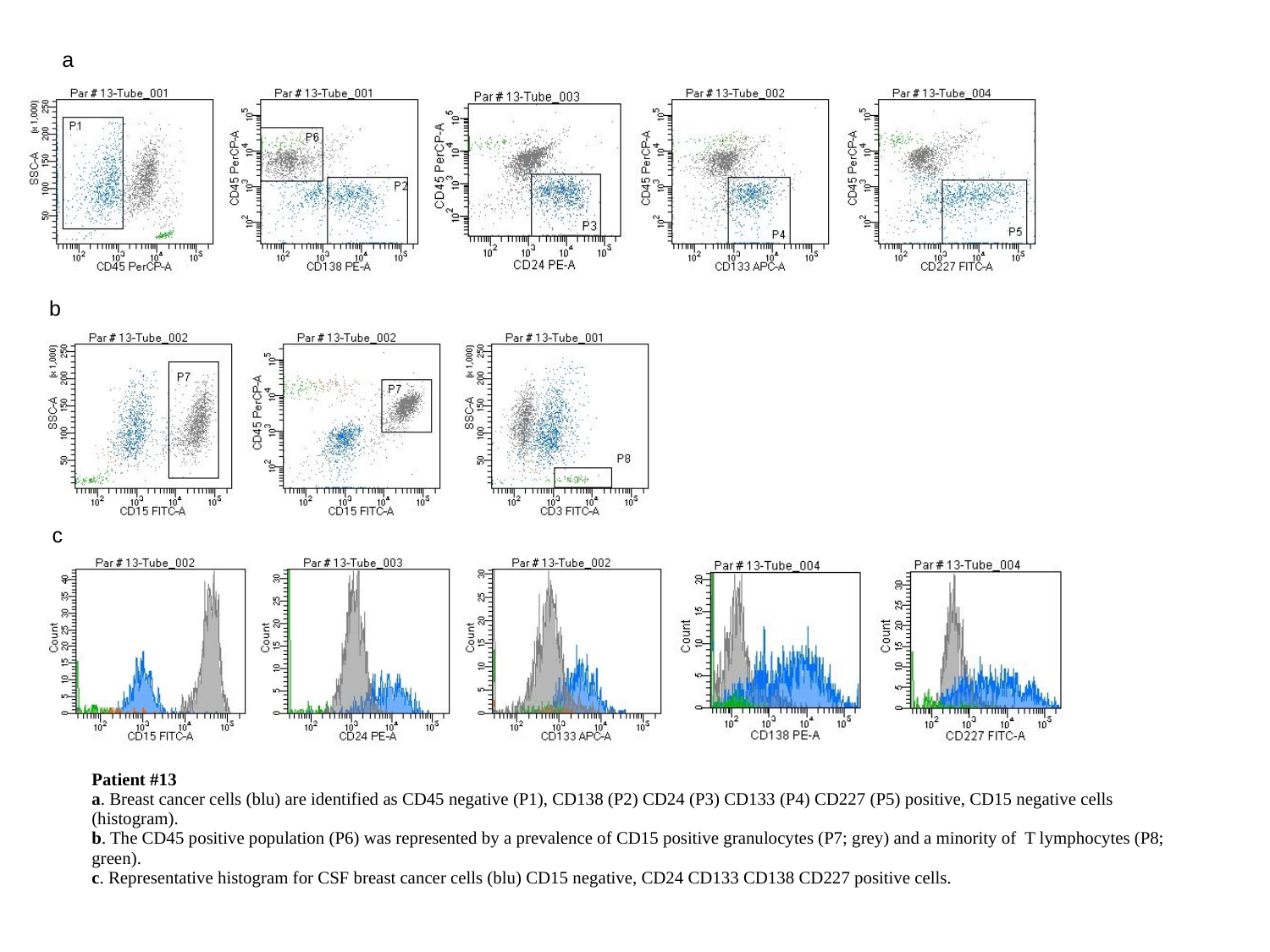

a
b
c
Patient #13
a. Breast cancer cells (blu) are identified as CD45 negative (P1), CD138 (P2) CD24 (P3) CD133 (P4) CD227 (P5) positive, CD15 negative cells (histogram).
b. The CD45 positive population (P6) was represented by a prevalence of CD15 positive granulocytes (P7; grey) and a minority of T lymphocytes (P8; green).
c. Representative histogram for CSF breast cancer cells (blu) CD15 negative, CD24 CD133 CD138 CD227 positive cells.
